# A marker gene-based method for identifying the cell-type of origin from single-cell RNA sequencing data

**DOI:** 10.1016/j.mex.2023.102196

**Published:** 2023-04-25

**Authors:** Nima Nouri, Giorgio Gaglia, Andre H. Kurlovs, Emanuele de Rinaldis, Virginia Savova

**Affiliations:** Precision Medicine and Computational Biology, Sanofi, 350 Water Street, Cambridge, MA 02141, USA

**Keywords:** scRNA-seq, Cell type, Annotation, Classification, Software, SARGENT - SignAtuRe-GEne cell aNnoTation

## Abstract

Single-cell RNA sequencing (scRNA-seq) experiments provide opportunities to peer into complex tissues at single-cell resolution. However, insightful biological interpretation of scRNA-seq data relies upon precise identification of cell types. The ability to identify the origin of a cell quickly and accurately will greatly improve downstream analyses. We present Sargent, a transformation-free, cluster-free, single-cell annotation algorithm for rapidly identifying the cell types of origin based on cell type-specific markers. We demonstrate Sargent's high accuracy by annotating simulated datasets. Further, we compare Sargent performance against expert-annotated scRNA-seq data from human organs including PBMC, heart, kidney, and lung. We demonstrate that Sargent retains both the flexibility and biological interpretability of cluster-based manual annotation. Additionally, the automation eliminates the labor intensive and potentially biased user annotation, producing robust, reproducible, and scalable outputs.

• Sargent is a transformation-free, cluster-free, single-cell annotation algorithm for rapidly identifying the cell types of origin based on cell type-specific markers.
• Sargent retains both the flexibility and biological interpretability of cluster-based manual annotation.
• Automation eliminates the labor intensive and potentially biased user annotation, producing robust, reproducible, and scalable outputs.

## Graphical abstract

**Sargent official logo.** Sargent is a transformation- and cluster-free cell-type annotation method that operates at individual cell resolution by applying a scoring system to scRNA-seq data based on sets of marker genes associated with cell types.

**Figure:**
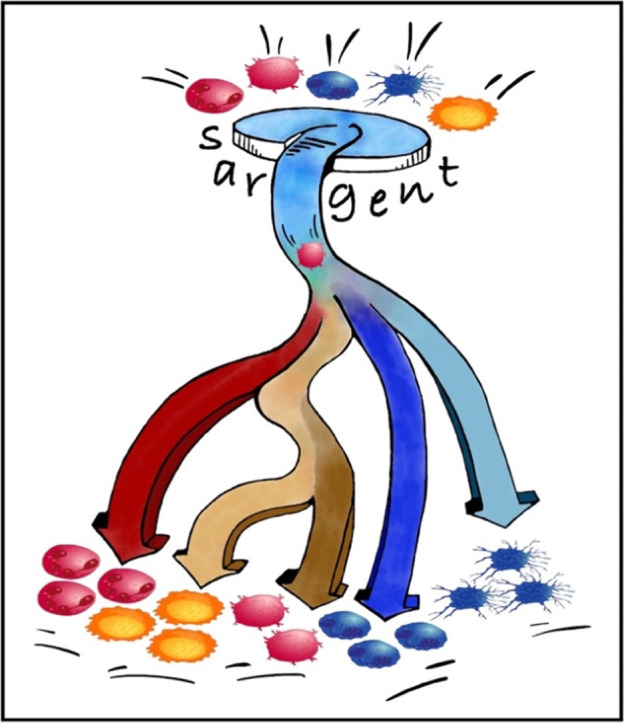

Specifications table

**Table:** 

Subject area:	Bioinformatics
More specific subject area:	*Single Cell RNA Sequencing*
Name of your method:	*SARGENT - SignAtuRe-GEne cell aNnoTation*
Name and reference of original method:	*Not applicable*
Resource availability:	1. *Sargent source code is publicly available at**github.com/Sanofi-Public/PMCB-Sargent* 2. *Data availability:* i. *Experiment data is publicly available from The Tabula Sapiens paper* (Tabula Sapiens Consortium 2022). ii. *Simulated data is available upon request*.

## Introduction

Single-cell RNA sequencing (scRNA-seq) has emerged as a powerful tool to characterize cell types and states in complex tissues and organisms at the single-cell level. However, accurate identification of cell types is imperative to comprehensively explore and exploit scRNA-seq data and to provide precise biological insights. Cell types are confounded by the phenotypic properties and diverse cellular states, making cell-type annotation a challenging task. Strategies have been so far proposed to overcome these challenges, which has resulted in four general avenues for annotating scRNA-seq data: (1) user-defined assignment through manual exploration of cell populations and marker expression, (2) automated annotation computational tools based on correlation strategies, (3) supervised classifier methods, and (4) gene set score-based annotators.

Manual annotation of single-cell clusters (the practice of manual investigation and labelling of cell clusters) using standard scRNA-seq data analysis tools, like Seurat [10,15] and Scanpy [17,20], is by far the most commonly used strategy, and it is rooted in identifying cell types through canonical marker genes. Current state-of-the-art pipelines start by preprocessing data and clustering cells into groups. These groups are then manually inspected for the expression of cell type-specific markers, based on which each group is assigned to a specific cell type. These marker features are either previously known from prior research or they are identified using differential expression analysis of the given cell group against the rest of the dataset. This strategy, however, is time-consuming, biased, and prone to error. Manual annotation requires the high-dimensional data to be transformed (normalized, scaled, batch corrected, etc.) for clustering and visualization. This preprocessing reshapes the data so that cells with comparable biological patterns of transcripts end up with similar transformed measurements, and hence fall closer to each other in the reduced-dimension gene-expression space. However, growing concerns have been raised that these transformations lead to unintended distortions when are used for clustering [6]. Furthermore, the cluster assignment becomes increasingly subjective as cells reach the cluster edges. Lastly, the lack of methodologies to assess the intrinsic sources of variability in high-dimensional data in a statistically rigorous manner often leads to overconfidence in the discovery of novel cell types [9].

Correlation-based annotation strategies have been developed to systematically assign cell types based on existing annotations from a “reference” or “benchmark” dataset. In this strategy, the algorithm takes in a dataset to be annotated as well as a previously annotated reference datasets, and it calculates how much each cell (or a cluster of cells) in the novel data correlates (feature expression) to a cell type in the reference dataset. Each cell or cluster is then annotated to the best correlated cell type found from the reference. Frequently used tools in this category are Seurat Reference Mapping [10,15], SingleR [4], and scmap-cluster [12]. However, correlation-based strategies have shortcomings because of the lack of comprehensive and high-quality reference datasets that researchers and bioinformaticians broadly agree upon. In addition, the annotated reference datasets are also subjective to investigator error and bias.

Supervised classification-based tools have been developed to predict cellular phenotypes in single-cell RNA-seq data using pre-trained classifiers [2,^5^,^13^,14]. These machine learning algorithms need to first be trained with annotated reference datasets (either single-cell or sorted bulk). Therefore, such supervised approaches face the additional challenge of requiring the reference dataset to reflect all the cell types expected in the exploratory datasets, which is often problematic in scRNA-seq studies, especially when rare cell subsets in complex tissues are involved (e.g., central nervous system cell types and specialized epithelial cells). Furthermore, marker-free machine learning models are intrinsically limited to cell types with broadly distinct transcriptional phenotypes and have limited sensitivity when subsets of cells with relatively few specific transcriptional characteristics need to be detected within broader subtypes (e.g., Th1 versus Th2 subsets of CD4 T-cells). Yet, exploring the abundance and characteristics of such rare but functionally important cells is one of the main applications of scRNA-seq.

Finally, score-based tools have been developed to classify scRNA-seq data according to assignment scores calculated for given gene set markers [1,22]. These methods are built upon the assumption that there is a bimodal distribution for each gene set, with the higher mode corresponding to the cell type of origin, and the lower mode corresponding to all the other cell types. However, for cell types with similar expression profiles, overlap between the two respective distributions is inevitable, meaning no threshold will adequately separate cells into those cell types. Therefore, a principal bottleneck in these methods is the ability to accurately identify the bimodality in the distribution. When they fail to identify a bimodal distribution, they rely on the separation of clusters of cells in a two-dimensional space, which makes them vulnerable to losing variation present in a high-dimensional input dataset. Other available score-based methods yet remain prone to unintended distortions due to data transformation and clustering requirements [11]. Another score-based tool is UCell which is primarily developed as a module scoring methodology [3]. UCell is notable for its ability to calculate scores based exclusively on the gene expression levels within individual cells, which makes it independent of dataset composition. However, while UCell bears this desirable feature, it lacks a definitive strategy to annotate each individual cell, as well as identifying unknown cell types.

We introduce Sargent, a novel cell type annotation method that works at individual cell resolution by performing a conceptually simple but decisive scoring system based on sets of marker genes associated with cell types. Sargent is transformation- and cluster-free which makes it immune from unwanted distortions caused by preprocessing steps and batch artifacts (the only required preprocessing step is the standard scRNA-seq quality control checks). Moreover, Sargent does not require a reference dataset which enables it to produce accurate and fast single-cell-type annotations. The only inputs required are a gene expression matrix and a list of gene sets.

We first describe the methodological and mathematical details of the Sargent algorithm. Next, we extensively benchmark the algorithm's performance on simulated data demonstrating its high sensitivity and specificity compared to the ground truth. Finally, we leverage a human multi-tissue scRNA-seq study from the Tabula Sapiens Consortium [18] to compare the automatic annotations from Sargent to the expert-annotated experimental data. The results highlight Sargent's ability to annotate single-cell types reliably and efficiently, making it a valuable tool in the field of single-cell transcriptomics.

## Method details

**Fig. 1:**
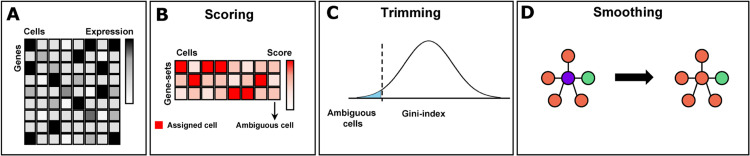
**An overview of the Sargent algorithm design.** (A) Input gene-by-cell expression matrix (number of genes × number of cells). The color shade indicates the expression level of genes (row) for each cell (column). (B) Schematic illustration of the scoring step. Gene-set-by-cell assignment-score matrix (number of gene sets × number of cells). The color shade indicates the assignment score of the gene set (row) for each cell (column). (C) Schematic illustration of the trimming step. Sargent uses the Gini index as a measure of dispersion to identify ambiguous calls. Cells with low values (outliers) of the Gini index distribution are annotated as unclassified. (D) Schematic illustration of the smoothing step. Sargent aggregates information from cells with similar genome-wide expression profiles (neighbors) to relabel the surrounded cell according to the neighbors' annotation consensus.

scRNA-seq annotation in Sargent is performed in three sequential steps: (1) scoring; (2) trimming: and (3) smoothing (Fig. 1). We discussed each step below.

### Scoring

The input to Sargent consists of a single-cell transcriptomics dataset (scRNA, or single nuclei [snRNA]), a set of cell types to be detected, and an associated gene set for each cell type (Fig. 1A). Sargent uses a score-based procedure to infer the cell type of origin for each cell based on the provided gene sets. First, for a given cell, it sorts non-zero expressed genes from high to low expression. Then, this ranked vector (of length N) is converted to a binary sequence (s) so that genes (g) that are included in a specific gene set (G) are substituted by 1, and 0 otherwise:

$$s={\left\{\begin{array}{c} 1:\;g_{n}\in G\\ 0:\;g_{n}\notin G \end{array}\right\}}_{1\leq n\leq N}.$$

Next, a partial cumulative sum is performed over the binary sequence up to each element “k” of the binary vector, followed by the sum over all generated sequential “1-to-k” partial sums, which results in the assignment-score S for the given cell:

$$S=\sum_{k=1}^{N}\sum_{n=1}^{k}s_{n}.$$

This process is performed over all cells and gene sets (total M gene sets), transforming an input gene-by-cell expression matrix (number of genes × number of cells) into a gene-set-by-cell assignment-score matrix (number of gene sets × number of cells). Finally, each individual cell is assigned to the cell type with the highest assignment-score (Fig. 1B):

$$\text{max}\;{\left\{S_{m}\right\}}_{1\leq m\leq M}.$$

The scoring method is cell-based and is therefore independent of the gene expression units.

### Trimming

To prevent misassignment when scores are calculated uniformly across cell types or unknown cell types (unspecified in the marker matrix) are present, Sargent annotate cells as unclassified (Fig. 1C). Sargent uses the Gini index [8] as a measure of dispersion to identify ambiguous calls. First, for each cell, the Gini index is calculated among its assignment scores, transforming the gene-set-by-cell assignment-score matrix to a distribution of indexes (∈[0,1]). Then, cells with an index which is both an outlier (μ−xσ; where μ is the mean, x is the confidence interval, and σ is the standard deviation) and less than 0.5 are called ambiguous. Statistically, a Gini index below 0.5 does not represent a severe dispersion among assignment scores, suggesting a poor annotation. Cells with such ambiguous calls remain unclassified. In addition, if a cell does not express any of the specified markers or gains an equal score across multiple cell-types, it will remain unclassified.

### Smoothing

The last step in cell annotation is k-nearest neighbor (kNN) smoothing (Fig. 1D). This step is designed to smoothen annotations by aggregating information from cells with similar genome-wide expression profiles (neighbors). Smoothing is performed by first identifying cells with a minimum of k nearest-neighbors. Then, if more than 50% of neighbors reach a consensus on their labels (i.e., the most frequent label of the nearest neighbors), the surrounded cell will be relabeled according to the neighbors' consensus. A kNN graph can be generated using state-of-the-art methods [10,19]. Smoothing is optional and runs once over all cells.

### Additional features and operational suggestions

Negative markers combined with positive markers can increase the specificity of cell type identification, reducing the likelihood of misclassification and improving the overall accuracy of the analysis [3]. By default, the signature genes are expected to be highly expressed in one cell type compared to all other cell types. However, depending on the underlying data, these canonical markers may not be enough to segregate cell types with similar expression profiles (e.g., sub-groups of T-cells in the human blood). When this occurs, genes that are expected *not* to be detected in a specific cell type (e.g., CD8A in CD4 T cells) can be utilized to improve segregation. Therefore, genes that are characteristically lowly expressed in one cell type compared to the other cell types are introduced as the “negative markers”. Sargent incorporates negative markers by rewarding cells that do not express these markers and penalizing them otherwise. Such procedure increases the dispersion among assignment scores, leading to a more trustworthy outcome. In addition, we note that the Sargent algorithm has been implemented so that markers (either positive or negative) can be shared across multiple gene sets, therefore providing significant flexibility for investigators to design comprehensive, decisive, and optimal gene sets.

Too many cell type-specific gene sets can make annotation more challenging, especially for very heterogeneous datasets. When the ontology of cell type is extremely granular, a common strategy is to perform sub-annotation by utilizing a hierarchy of established cell types [4]. More specifically, data could undergo a first round of annotation at an intermediate hierarchy level (i.e., with broad cell type definitions). Subsequently, each group can be treated as a new separate dataset and annotated further with other gene sets. By focusing on just a subset of the data, the granularity will increase, and novel cell subtypes can be explored efficiently.

The list of gene sets to explore on the cells is one of the fundamental building blocks in Sargent. Therefore, it is imperative that investigators have a detailed understanding of cell types and associated markers they expect to observe in the tissue under investigation. A list of tissue-specific markers could come from a variety of sources. The two largest databases for cell type markers are available at CellMarker [23] and PanglaoDB [7]. Expert-annotated data are also available from databases like The Tabula Sapiens [18], which could be analyzed to define gene signatures. Alternatively, signatures could also be flexibly defined from investigators’ own experiments like CITE-seq [16] or cross-validation experiments.

Sargent can be used as a stand-alone tool or as complementary to the other supervised methods. For instance, commonly used scRNA-seq analysis workflows like Seurat [10,15] may be used to identify segregated cell populations and apply Sargent to validate and refine the granularity of cell type annotation.

## Method validation

### Validation of the sargent algorithm using simulated scRNA-seq data

**Fig. 2:**
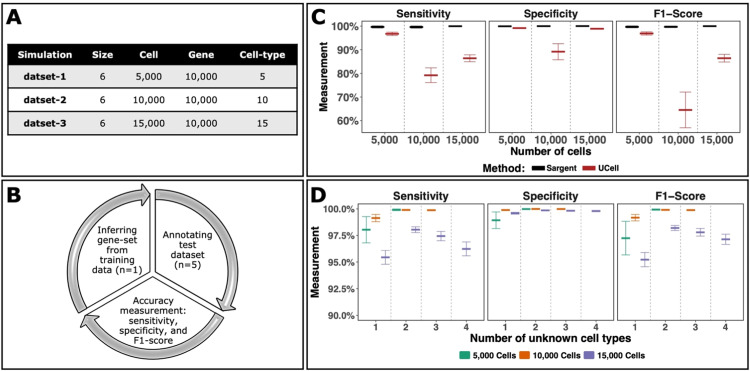
**Sargent identifies cell types of origin with high confidence on simulated data.** (A) Overview of 18 simulated datasets generated by the Splatter R package. (B) Schematic illustration of the benchmarking procedure. (C) Annotation of single cells using the Sargent and UCell algorithms. Performance was assessed by calculating sensitivity, specificity, and the F-1 score for simulated datasets with 5k, 10k, or 15k cells. (D) Sargent performance with under-specification of the marker gene sets. Sargent performance was assessed by calculating sensitivity, specificity, and the F-1 score where one to four cell types were removed from the training data. Marker genes were inferred without knowledge of the removed cell types.

We first benchmarked the performance of the Sargent on simulated data, where cell types are known a priori. Specifically, we used Splatter R package (with default parameters) [21] to create three sets of simulated scRNA-seq data of increasing size and complexity: (1) six datasets with 5,000 cells divided into five cell-types; (2) six datasets with 10,000 cells divided into ten cell types; (3) six datasets with 15,000 cells divided into 15 cell types. The proportion of cells in each cell-type group were randomly sampled from a uniform distribution. Each simulated dataset was composed of 10,000 genes (Fig. 2A).

Sargent requires cell type-specific markers as the input. To infer them from simulated data we designed a systematic approach. First, we selected one simulated dataset as the training dataset (1 training + 5 testing). We inferred marker gene sets from the training data by performing differential expression analysis using Wilcoxon rank-sum test (“FindAllMarkers” function from Seurat R package with a minimum fraction of 0.1 cells expressing a given gene and at least 0.5-fold difference (log-scale) between the two groups of cells). The 100 top-ranked marker genes for each cell type were used as the input for annotation of the remaining five testing datasets. This approach provided a benchmark of 30 trials for each set of simulated data (Fig. 2B).

The performance was quantified using the measures of sensitivity (SEN), specificity (SPC), and F1-score by the fractions TP/(TP+FN), TN/(TN+FP), and 2×TP/(2×TP+FP+FN), respectively, where:

1. True positive (TP) is defined by the number of cell type-related pairs that are correctly identified,
2. False positive (FP) is defined by the number of unrelated cell pairs that are incorrectly identified as cell type-related,
3. True negative (TN) is defined by the number of unrelated cell pairs that are correctly identified as unrelated, and
4. False negative (FN) is defined by the number of cell type-related pairs that are incorrectly identified as unrelated.

We found that Sargent inferred the simulated cell type assignments with average sensitivity, specificity, and F1-score of above 99% across all trials (Fig. 2C). Next, we sought to compare the performance of Sargent with UCell [3]. Given that UCell lacks the capability to annotate individual cells definitively, we opted to assign each cell to the cell type that obtained the highest score according to UCell's scoring system. To ensure a fair comparison, we used the same simulated dataset and gene sets. Our analysis revealed that Sargent consistently outperformed UCell in terms of key performance metrics, including sensitivity, specificity, and F1-score (Fig. 2C).

We next sought to examine robustness of Sargent performance in discovering novel cell types (i.e., unclassified cell types). This task was performed by omitting a subset of the marker gene sets (under-specification of the marker gene sets) and applying Sargent to retrieve the missing cell types. We randomly chose one training dataset, then we randomly removed between 1 and 4 cell types from the training data: 1–2 cell types from data with five cell types; 1–3 cell types from data with 10 cell types; and 1–4 cell types from data with 15 cell types. Then, we inferred marker gene sets from the training data by performing differential expression analysis as discussed above. Last, we benchmarked Sargent's performance among the remaining simulated data. We repeated this procedure 100 times for each cell type removal. We note that cell types were removed prior to marker gene selection to ensure that marker genes were being selected with no knowledge of unknown cell types. We found that Sargent inferred the unknown cell types with average sensitivity, specificity, and F1-score values of above 95.0% across all trials (Fig. 2D). It should be noted that conducting similar analysis using UCell [3] was not viable as UCell is not equipped to identify unknown cell types.

We further note that Sargent is not sensitive to the inclusion of marker gene sets for cell types that are not present in the dataset (over-specification of the marker gene sets). Since Sargent is a single-cell-based algorithm (i.e., cells do not compete against each other), the inclusion of a marker set for which no cells are found does not affect the score of the other marker gene sets, and hence it does not impact the annotation quality of the cell types present in the dataset.

### Validation of the sargent algorithm using experimental scRNA-seq data

**Fig. 3:**
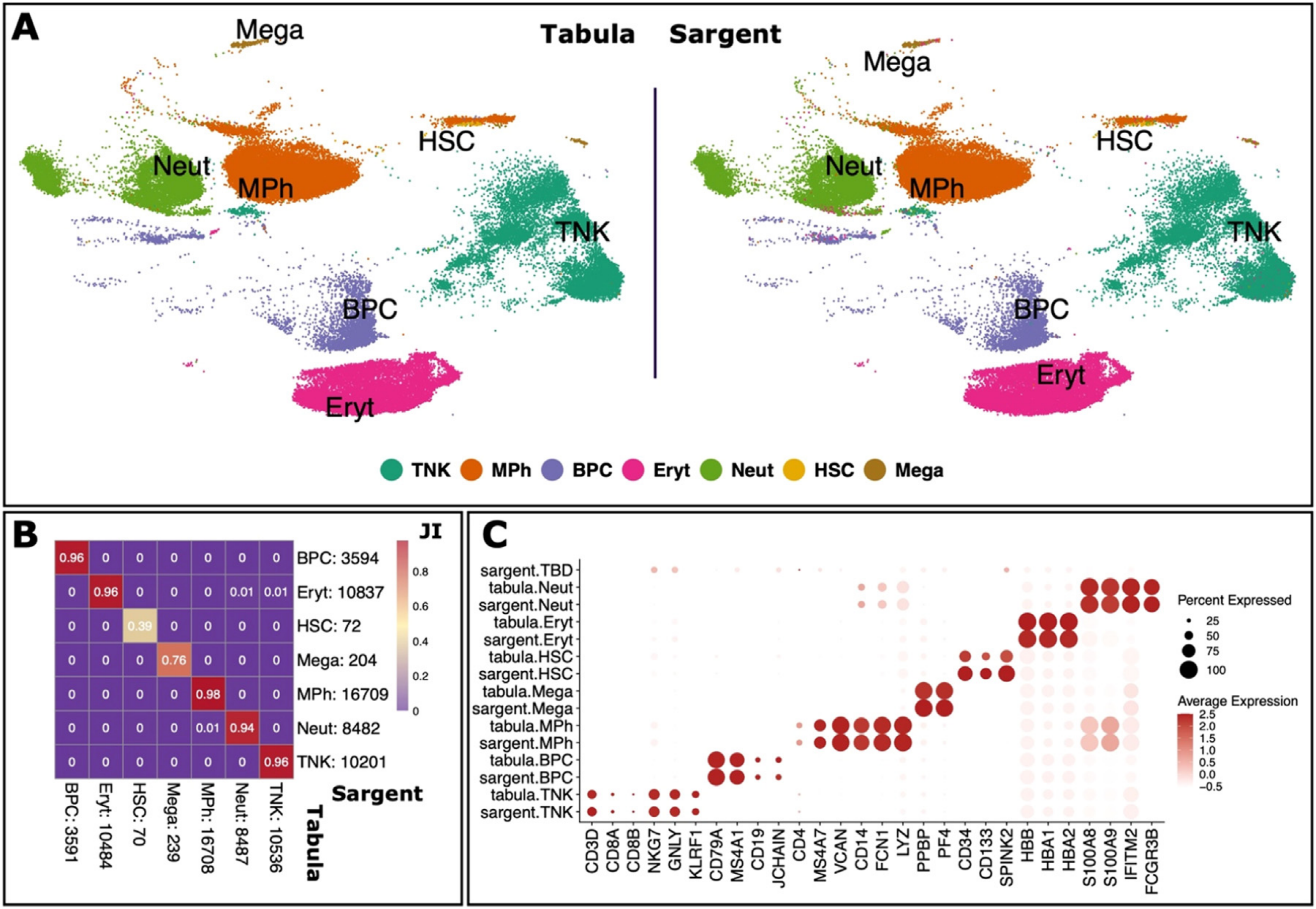
**Sargent identifies the cell-types of origin with high confidence in human PBMCs.** (A) t-SNE plot showing the cluster-based manual annotation of cells from the original Tabula publication (left panel) and the cell types assigned by Sargent (right panel). (B) Heatmap comparing the number of cells annotated by experts (column) versus Sargent (row). Color represents the Jaccard Index calculated as the ratio of intersection over union between each pair of cell types. Value next to each cell-type label indicates the number of annotated cells. (C) Dot-plot comparing the expression level of selected markers between expert-annotated cell types versus the Sargent cell types.

Along with simulated data, we investigated the performance of Sargent by annotating experimental scRNA-seq data from multiple human tissues, including PBMC, heart, kidney, and lung, from published Tabula Sapiens Consortium [18]. We first extracted 50,115 peripheral blood mononuclear cells (PBMCs) and classified them using a hierarchy of known immune cell-types. We first created marker gene sets specifying cell types at a moderate immunophenotype granularity, namely: T cells and NK cells (TNK), monocytes and macrophages (MPh), B and plasma cells (BPC), neutrophils (Neut), erythrocytes (Eryth), megakaryocytes (Mega), and hematopoietic stem cells (HSC, Supplementary File S1). Sargent assigned cells to the correct type (the expert-annotated type), with 98% accuracy across all cell types (Fig. 3A). High assignment accuracy was also demonstrated by the Jaccard Index (JI): 98% among MPhs; 96% among TNKs, BPCs, and Eryts; 94% among Neuts; and 76% among Megas (Fig. 3B). A relatively lower JI was achieved among HSCs: 39%. However, when we explored the expression level of the HSCs’ canonical markers, CD34, CD133, and SPINK2, we observed that cells annotated by Sargent are more likely to be HSCs than the manually annotated cells (Fig. 3C). Only 16 out of more than 50,000 cells were not assigned to a cell type by Sargent as they did not have shown any expression of all the immunophenotypes’ canonical markers (Fig. 3C).

**Fig. 4:**
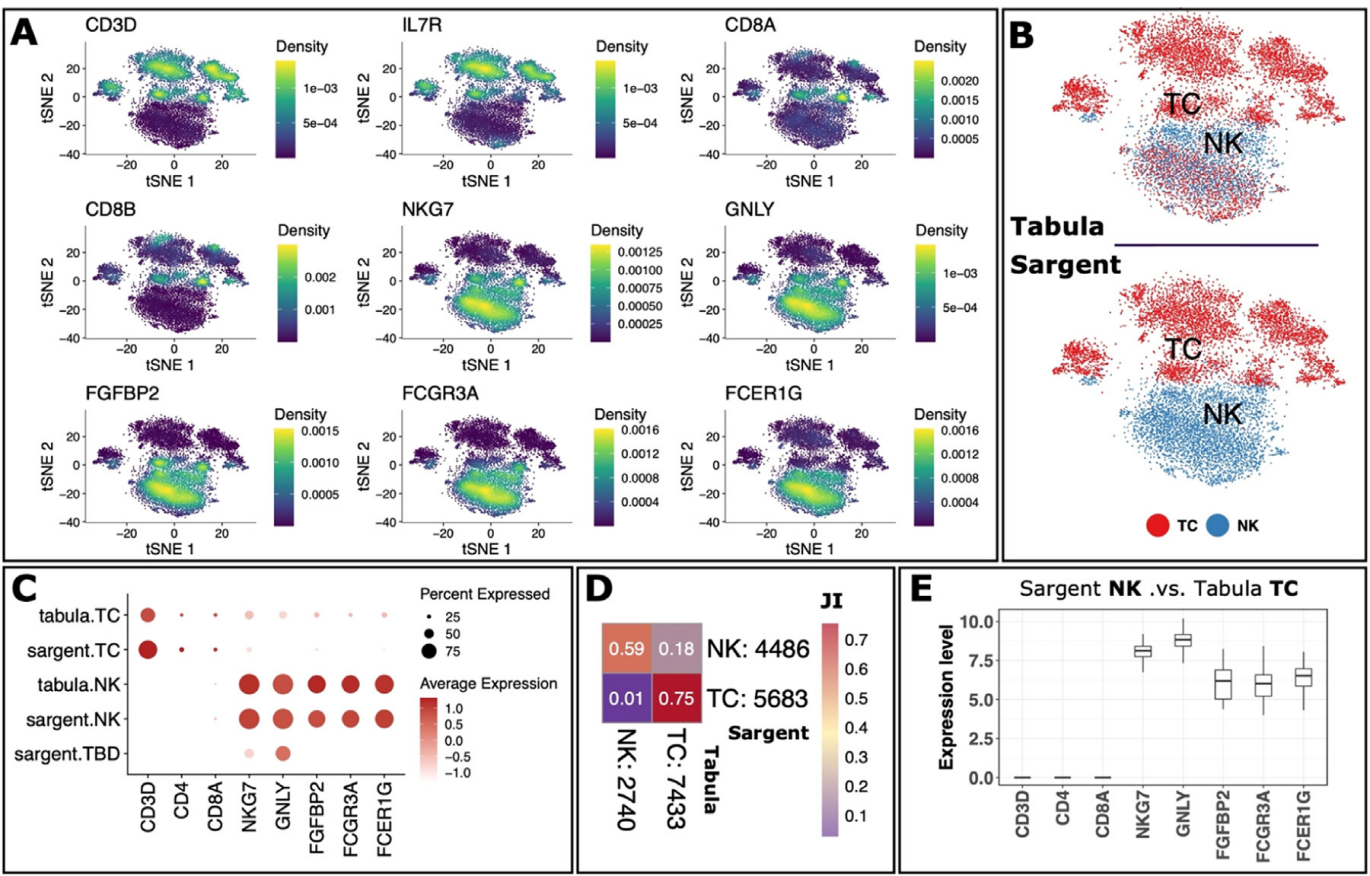
**Sargent identifies T and natural killer cells with high confidence in human PBMCs.** (A) t-SNE plots representing expression levels of selected genes. (B) t-SNE plot showing the manual expert cluster-based annotation of cell types from the original Tabula publication (top panel) and the cell types assigned by Sargent (bottom panel). (C) Dot-plot comparing the expression level of selected markers between expert-annotated cell types versus the Sargent cell types. (D) Heatmap comparing the number of cells annotated by experts (column) versus Sargent (row). Color represents the Jaccard Index calculated as the ratio of intersection over union between pairs of cell types. Value next to each cell-type label indicates the number of annotated cells. (E) Expression level of selected markers for cells annotated as NK by Sargent and T by Tabula.

We next evaluated Sargent's performance by increasing the granularity of annotated cell types within the PBMCs. We first retrieved 10,173 cells that were jointly annotated as TNK by Sargent and Tabula. Next, we curated a list of T and NK (natural killer) cells canonical markers (Supplementary File S1 and Fig. 4A). Sargent annotated 5,683 T cells and 4,486 NK cells with a clear separation in t-SNE space (Fig. 4B), with only 4 cells remaining unlabeled (Fig. 4C). The Jaccard Index similarity between Sargent and Tabula was 75% among T cells and 59% among NK cells (Fig. 4D). We identified a mixed population (18% JI) of 1,795 cells (17% of TNKs) with Sargent NK annotation and Tabula T annotation (Fig. 4D). We explored this population to determine which annotation was more plausible. We examined the expression level of T-cell canonical markers including CD3D, CD4, and CD8A. Our inspection showed that this mixed population did not express the T-cell canonical markers (Fig. 4E). In contrast, they highly expressed cytotoxic markers like NKG7, GNLY, FGFBP2, FCGR3A, and FCER1G (Fig. 4E). We hence concluded that it is more plausible for this population to be annotated as NK cells (as annotated by Sargent), as opposed to T cells (as annotated by Tabula).

**Fig. 5:**
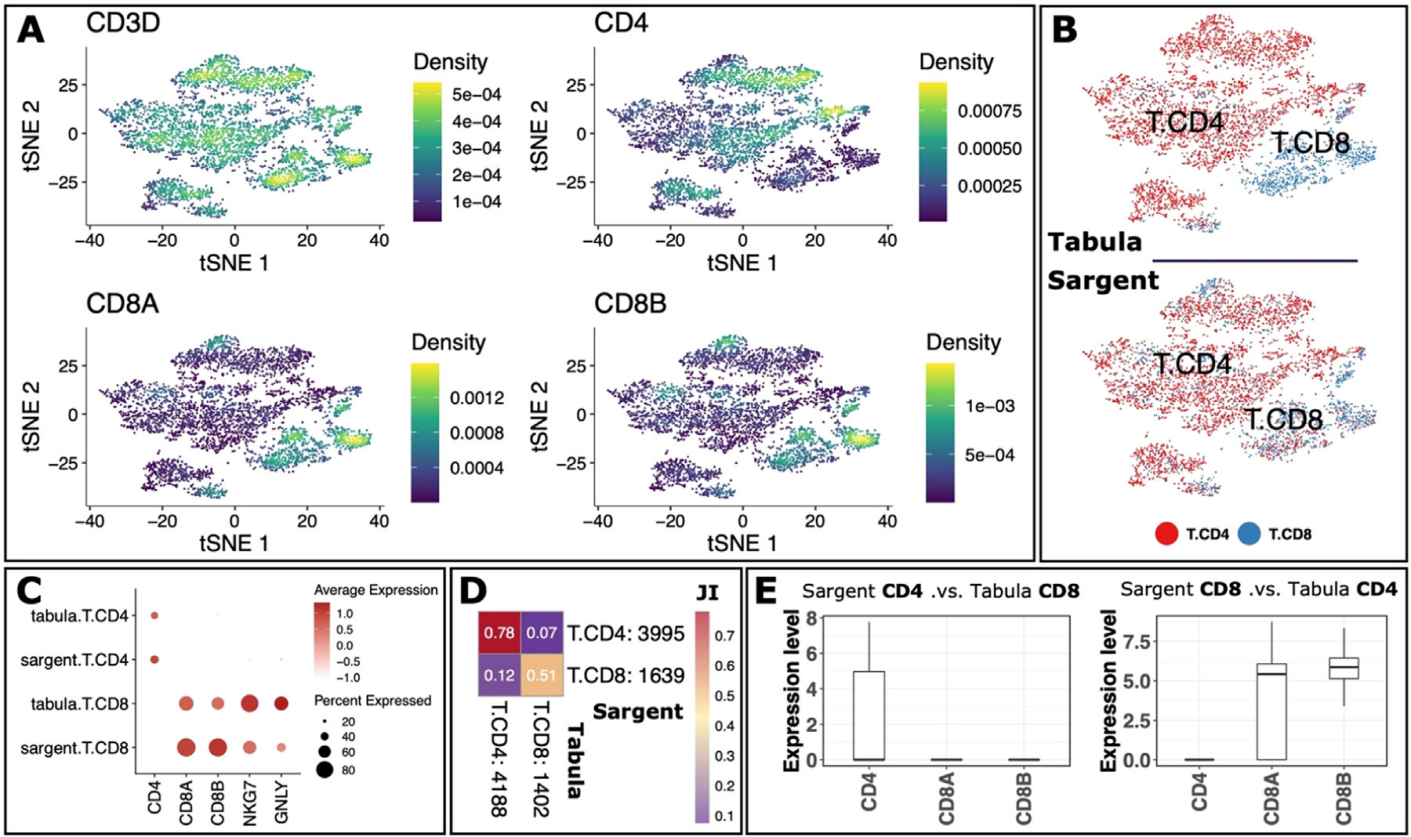
**Sargent identifies CD4+ T and CD8+ T cells with high confidence in human PBMCs.** (A) t-SNE plots representing expression level of selected genes. (B) t-SNE plot showing the manual expert cluster-based annotation of cell types from the original Tabula publication (top panel) and the cell types assigned by Sargent (bottom panel), (C) Dot-plot comparing the expression level of selected markers between expert-annotated cell types versus the Sargent cell types. (D) Heatmap comparing the number of cells annotated by expert (column) versus Sargent (row). Color represents the Jaccard Index calculated as the ratio of intersection over union between pairs of cell types. Value next to each cell-type label indicates the number of annotated cells. (E) Expression level of selected markers for cells annotated as CD4+ by Sargent and CD8+ by Tabula (left panel), and cells annotated as CD8+ by Sargent and CD4+ by Tabula (right panel).

We next increased the resolution of the analysis by focusing only on T cells. We first retrieved 5,634 cells that were jointly annotated as T cells by Sargent and Tabula. We then curated a list of CD4+ and CD8+ T*-*cell canonical positive and negative markers (Supplementary File S1 and Fig. 5A). Sargent annotated 3,995 CD4+ T and 1,639 CD8+ T cells (Fig. 5B and C). The Jaccard Index similarity between Sargent and Tabula was 78% among CD4+ T and 51% among CD8+ T cells (Fig. 5D). In addition, we observed a mixed population (17% of TCs) among Sargent and Tabula annotated cells comprised of 376 cells (7% JI) annotated as CD4+ T by Sargent but CD8+ T cells by Tabula, and 605 cells (12% JI) vice versa (Fig. 5D). We explored these two groups to determine the more plausible annotation. We examined the expression level of T-cell canonical markers, CD4, CD8A, and CD8B. Our inspection showed that the Sargent-specific CD4+ T cells expressed the CD4 marker, and not CD8A and CD8B (Fig. 5E: left-panel). In contrast, the Tabula-specific CD8+ T cells did not express CD8A and CD8B markers. Furthermore, Sargent-specific CD8+ T cells expressed CD8A and CD8B, and not CD4 (Fig. 5E: right-panel), while Tabula-specific CD4+ T cells expressed a relatively high level of CD8A and CD8B, but not CD4. Thus, this inspection revealed that Sargent's annotation was more plausible than Tabula's for the CD4+ and CD8+ subpopulations.

**Fig. 6:**
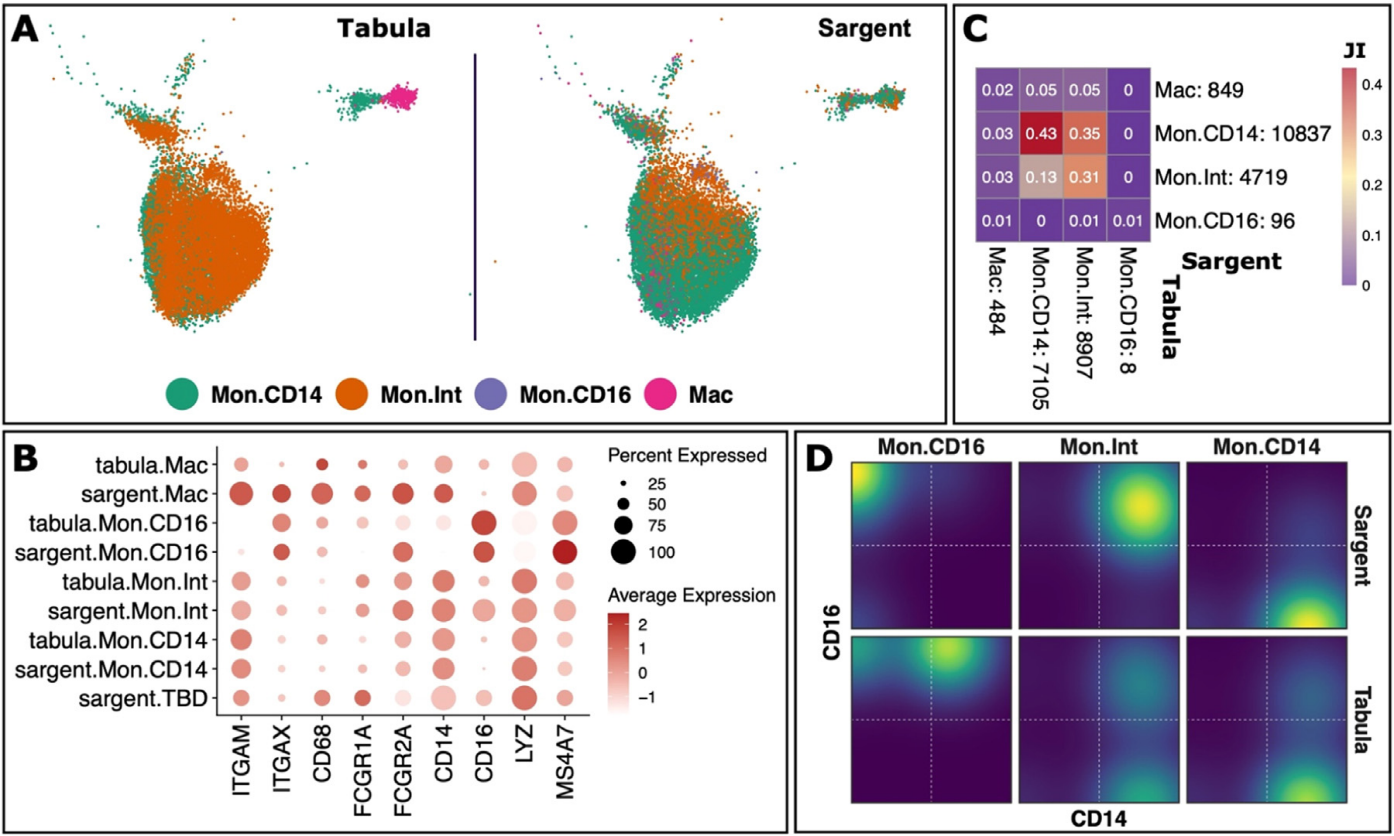
**Sargent identifies monocytes and macrophages with high confidence in human PBMCs.** (A) t-SNE plot showing the manual expert cluster-based annotation of cell types from the original Tabula publication (left panel) and the cell types assigned by Sargent (right panel). (B) Dot-plot comparing the expression level of selected markers between expert-annotated cell types versus the Sargent cell types. (C) Heatmap comparing the number of cells annotated by expert (column) versus Sargent (row). Color represents the Jaccard Index calculated as the ratio of intersection over union between pairs of cell types. Value next to each cell-type label indicates the number of annotated cells. (D) Expression level of CD14 (x-axis) and CD16 (y-axis) in cells annotated by Sargent (top panels) and Tabula (bottom panels) as non-classical monocytes (Mon.CD16), intermediate monocytes (Mon.Int), and classical monocytes (Mon.CD14).

We next switched our focus to macrophages and monocytes. Specifically, we benchmarked Sargent's performance by annotating macrophages and the three major monocyte populations: classical (CD14+CD16-), intermediate (CD14+CD16+), and non-classical (CD14-CD16+). We first retrieved 16,504 cells that were jointly annotated as MPh by both Sargent and Tabula. We then compiled a list of canonical macrophage and monocyte markers (Supplementary File S1). Sargent annotated 894 macrophages, 10,837 classical monocytes, 4,719 intermediate monocytes, 96 non-classical monocytes, and 3 unlabeled (Fig. 6A and B). The similarity between Sargent and Tabula annotation measured by Jaccard Index indicated a poor agreement (Fig. 6C) and, as for T and NK cells, we sought to determine which annotation was more plausible. We first examined the expression level of canonical macrophage markers including ITGAM, ITGAX, CD68, FCGR1A, and FCGR2A. Our inspection showed that macrophages annotated by Sargent expressed a higher level of the canonical markers than Tabula-annotated macrophages (Fig. 6B). Further inspection in the monocyte populations showed that Sargent achieved a clear segregation among classical, intermediate, and non-classical monocytes (Fig. 6D). In contrast, cells annotated by Tabula showed a mixed population, indicating a faulty annotation. Thus, these analyses revealed that cells annotated by Sargent are more likely to be macrophages or monocytes than manually annotated cells.

**Fig. 7:**
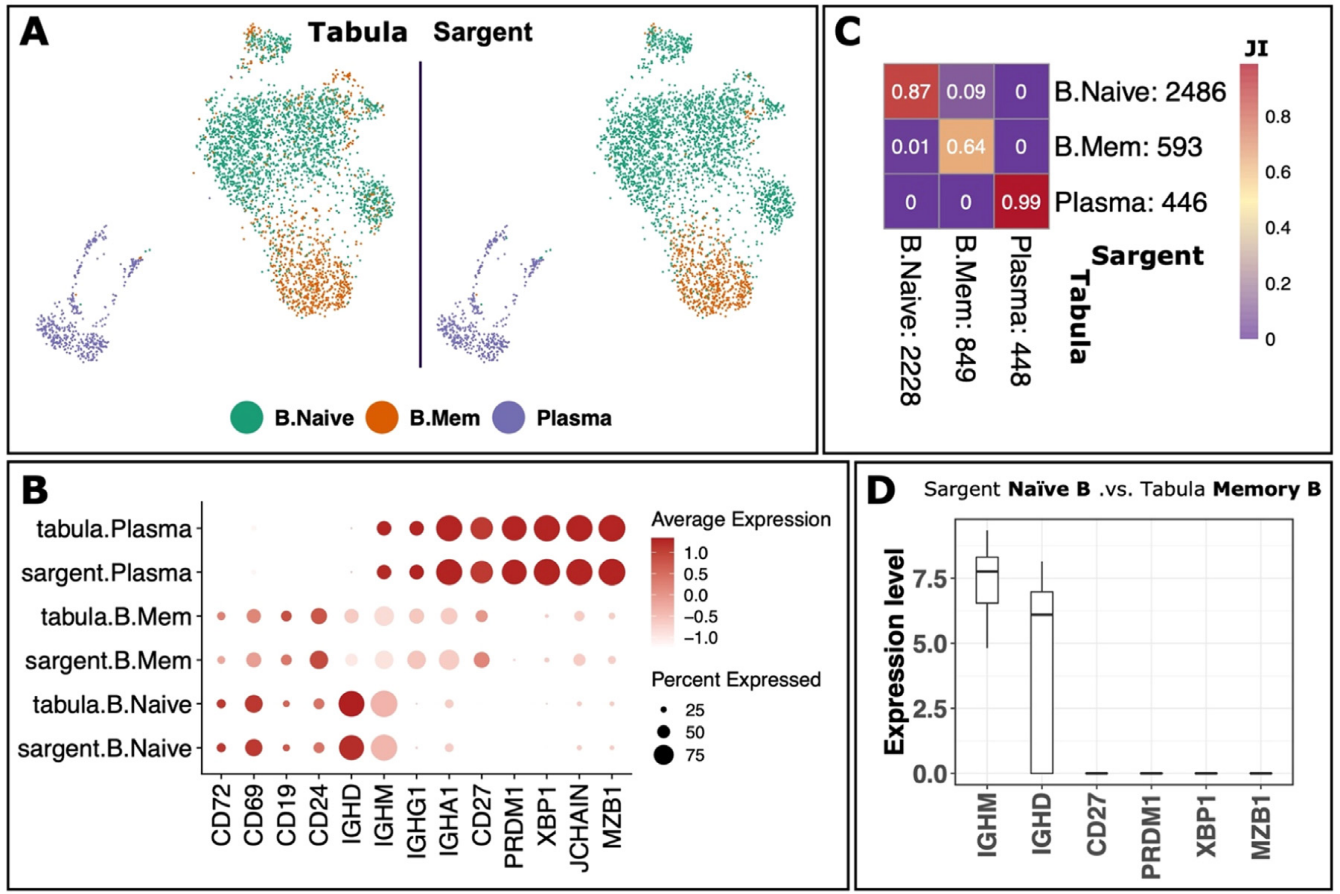
**Sargent identifies B and plasma cells with high confidence in human PBMCs.** (A) t-SNE plot showing the manual expert cluster-based annotation of cell types from the original Tabula publication (left panel) and the cell types assigned by Sargent (right panel). (B) Dot-plot comparing the expression level of selected markers between expert-annotated cell types versus the Sargent cell types. (C) Heatmap comparing the number of cells annotated by experts (column) versus Sargent (row). Color represents the Jaccard Index calculated as the ratio of intersection over union between pairs of cell types. Value next to each cell-type label indicates the number of annotated cells. (D) Expression level of selected markers for cells annotated as Naïve B by Sargent and Memory B by Tabula.

We next benchmarked Sargent's performance on the BPC population comprised of naïve B cells (NBCs), memory B cells (MBCs), and plasma cells (PCs). We retrieved 3,525 cells that were jointly annotated as BPC by Sargent and Tabula. We then curated a list of canonical markers highlighting memory, naïve, and plasma states (Supplementary File S1). Sargent annotated 2,486 NBCs, 593 MBCs, and 446 PCs (Fig. 7A and B). The Jaccard Index similarity between Sargent and Tabula annotation was 87% similarity among NBCs, 64% similarity among MBCs, and 99% PCs (Fig. 7C). We identified a mixed population (9% JI) of 284 cells (8% of BPCs) comprised of Sargent annotated NBCs and Tabula annotated MBCs (Fig. 7C). When we examined the expression level of canonical BC markers including naïve markers IGHM and IGHD, and activation marker CD27, this population highly expressed IGHM and IGHD and lacked the expression of CD27 (Fig. 7D). Thus, Sargent's annotation of this population as NBCs is more likely to be correct.

**Fig. 8:**
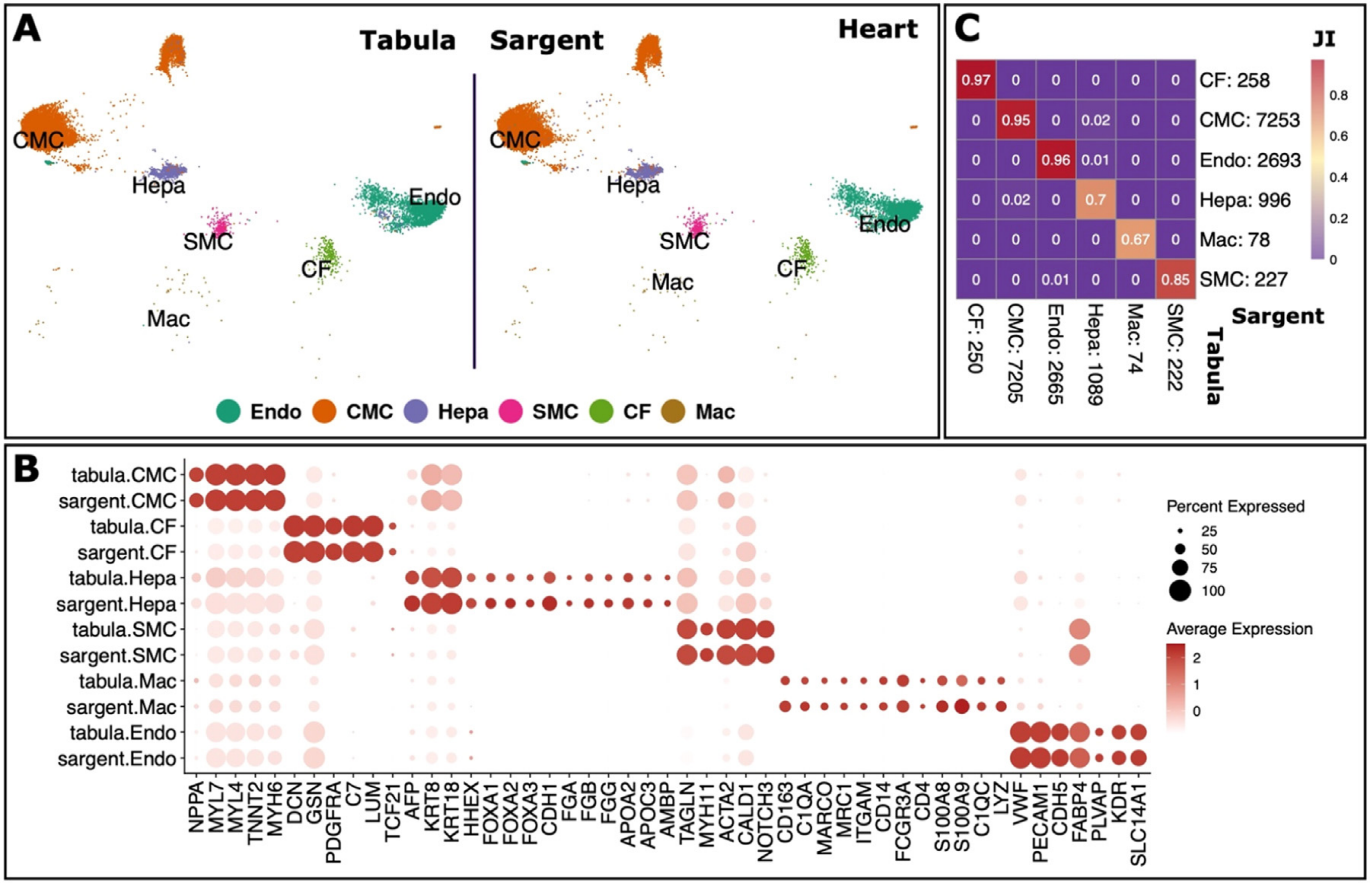
**Sargent identifies the cell-types of origin with high confidence in human Heart.** (A) t-SNE plot showing the manual expert cluster-based annotation of cell types from the original Tabula publication (left panel) and the cell types assigned by Sargent (right panel). (B) Dot-plot comparing the expression level of selected markers between expert annotated cell types versus the Sargent cell types. (C) Heatmap comparing the number of cells annotated by expert (column) versus Sargent (row). Color represents the Jaccard Index calculated as the ratio of intersection over union between pairs of cell types. Value next to each cell-type label indicates the number of annotated cells.

We next tested Sargent's performance in assigning cell types of additional three human tissues (heart, kidney, and lung) as compared to the manual cell type annotations from the Tabula Sapiens study. The manual annotation of the human heart tissue (11,505 cells) comprised six cell types including: cardiac fibroblasts (CFs), cardiac muscle cells (CMC), endothelial cells (Endo), hepatocytes (Hepa), macrophages (Mac), and smooth muscle cells (SMC). We compiled a set of marker genes from literature to recognize these cell types in the dataset (Supplementary File S1). Sargent assigned cells with 96% similarity with the manually annotated cells over all six heart tissue cell types (Fig. 8A and B). High assignment similarity between Sargent and the manual annotation was shown at individual cell type level as measured by Jaccard Index: 97% for CFs; 96% for Endo cells; 95% for CMCs; 85% for SMCs; 70% similarity Hepa cells; and 67% for Macs (Fig. 8C).

**Fig. 9:**
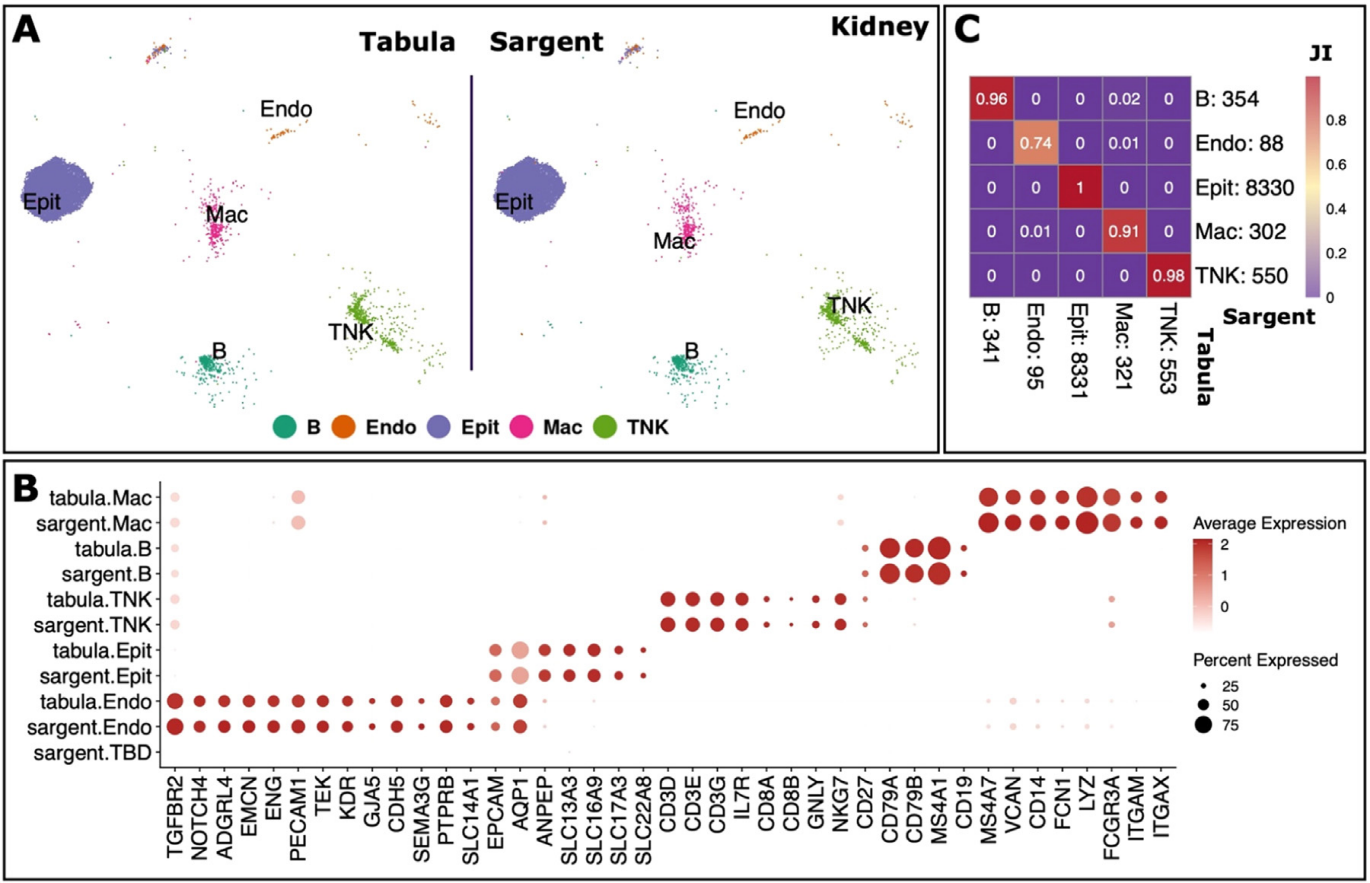
**Sargent identifies the cell-types of origin with high confidence in human Kidney.** (A) t-SNE plot showing the manual expert cluster-based annotation of cell types from the original Tabula publication (left panel) and the cell types assigned by Sargent (right panel). (B) Dot-plot comparing the expression level of selected markers between expert annotated cell types versus the Sargent cell types. (C) Heatmap comparing the number of cells annotated by expert (column) versus Sargent (row). Color represents the Jaccard Index calculated as the ratio of intersection over union between pairs of cell types. Value next to each cell-type label indicates the number of annotated cells.

Next, we switched our focus to human kidney tissue, which included 9,461 cells. The original annotation comprised of 5 cell types: B cells (B), endothelial cells (Endo), Epithelial cells (Epit), macrophages (Mac), and T and natural killer cell population (TNKs), for each of which we compiled a set of marker genes (Supplementary File S1). Sargent was again able to achieve a high similarity (99%) with the manually annotated cells over all the cell types (Fig. 9A and B), with only 17 cells remaining unlabeled, and hence with no strong indication among listed markers (Fig. 9B). High assignment similarity between Sargent and the manual annotation was demonstrated by the high Jaccard Index: 100% for Epit cells; 98% for TNKs; 96% for B cells; 91% for Macs; and 74% for Endo cells (Fig. 9C).

**Fig. 10:**
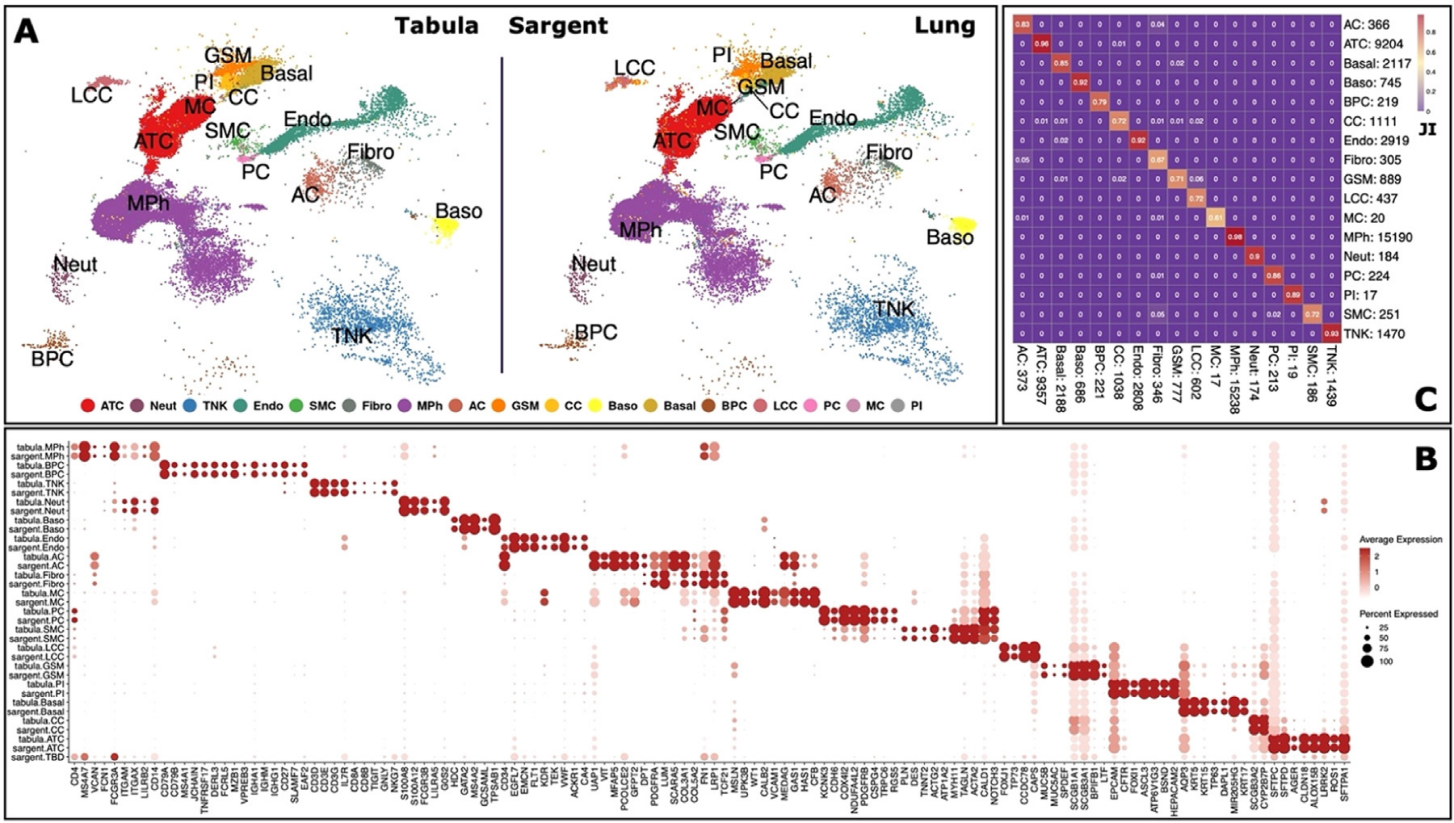
**Sargent identifies the cell-types of origin with high confidence in human Lung.** (A) t-SNE plot showing the manual expert cluster-based annotation of cell types from the original Tabula publication (left panel) and the cell types assigned by Sargent (right panel). (B) Dot-plot comparing the expression level of selected markers between expert annotated cell types versus the Sargent cell types. (C) Heatmap comparing the number of cells annotated by expert (column) versus Sargent (row). Color represents the Jaccard Index calculated as the ratio of intersection over union between pairs of cell types. Value next to each cell-type label indicates the number of annotated cells.

As our last test, we examined Sargent's performance on the complex human lung tissue (35,682 cells from Tabula Sapiens study). The original annotation included 17 main cell types: adventitial cells (AC), alveolar type 1 and type 2 cells (ATC), basal cells (Basal), basophils (Baso), B and Plasma cells (BPC), club cells (CC), endothelial (Endo), fibroblasts (Fibro), goblet, serous, and mucous cells (GSM), lung ciliated cells (LCC), mesothelial cells (MC), monocytes, dendritic cells, and macrophages (MPh), neutrophils (Neut), pericyte cells (PC), pulmonary ionocytes (PI), smooth muscle cells (SMC), CD4+ T cells, CD8+ T cells, and natural killer cells (TNK). We compiled a set of marker genes from literature to detect these cell types in the dataset (Supplementary File S1). Sargent was able to achieve a high similarity (96%) with the manually annotated cells over all the cell types, and only 14 cells remaining unlabeled (Fig. 10A and B). In addition, the high assignment similarity between Sargent and manual annotation was maintained at individual cell type level (Fig. 10C).

### Computationally efficiency

Computational efficiency is an important property considering the recent growth in the size of typical scRNA-seq datasets. Sargent is computationally inexpensive due to its single cell-level implementation; the algorithm's runtime grows linearly based on the input number of cells (O(n)), rather than with the size of the input gene expression matrix (quadratic growth – O(n^2^)). We recorded the time it took for Sargent to run using one single core with a 3.1 GHz processor and 128 GB Memory. Sargent annotation of 15,000 simulated cells (the largest simulated data used in this study) took less than 2 min. In addition, the annotation of ∼27k ± 20k (mean ± standard deviation) experimental cells (the average data size used in this study) took ∼2.3 ± 1.7 min.

## Conclusion

Identifying the cell type of origin for single cells is a key step in scRNA-seq data analysis. In this study, we developed Sargent (SignAtuRe-GEne cell aNnoTation), a score-based method that uses previously established gene sets of cell type-specific markers to assign cell identities. Sargent's scoring system is applied individually to each cell, and therefore its performance is independent of gene expression units and data transformations, including normalizations. Furthermore, since the cells are evaluated individually, Sargent's algorithm is highly scalable and can easily be applied to large datasets while maintaining the linear computational efficiency. Sargent is immune from data composition, processing, and batch artifacts, as it is both transformation- and cluster-free. Sargent is also capable of incorporating both positive and negative marker genes for cell type annotations. We demonstrated Sargent's accuracy in multiple scenarios. When applied to simulated data, Sargent showed high sensitivity and specificity. In addition, Sargent's showed a robust performance in discovering novel cell types (unclassified cells that are not represented within the gene sets). If validated as truly novel, the associated gene markers can be identified and used to design a more comprehensive gene set. Finally, using previously annotated experimental data from multiple human tissues, we demonstrated that Sargent's performance matches and exceeds the manual annotation by identifying more plausible cell types based on canonical markers. The Sargent package is available at github.com/Sanofi-Public/PMCB-Sargent as an R package, and it includes vignettes for both cell type annotation and seamless integration with Seurat object.

## CRediT authorship contribution statement

**Nima Nouri:** Conceptualization, Methodology, Software, Writing – original draft, Writing – review & editing. **Giorgio Gaglia:** Conceptualization, Methodology, Writing – review & editing. **Andre H. Kurlovs:** Software, Writing – review & editing. **Emanuele de Rinaldis:** Supervision. **Virginia Savova:** Conceptualization, Methodology, Writing – review & editing, Supervision.

## Declaration of Competing Interest

The authors declare the following financial interests/personal relationships which may be considered as potential competing interests: The authors are employees of Sanofi US.

## Data Availability

Data will be made available on request.

## Appendix Supplementary materials

Image, application 1
